# Predictive Role of the Apparent Diffusion Coefficient and MRI Morphologic Features on IDH Status in Patients With Diffuse Glioma: A Retrospective Cross-Sectional Study

**DOI:** 10.3389/fonc.2021.640738

**Published:** 2021-05-13

**Authors:** Jun Zhang, Hong Peng, Yu-Lin Wang, Hua-Feng Xiao, Yuan-Yuan Cui, Xiang-Bing Bian, De-Kang Zhang, Lin Ma

**Affiliations:** ^1^ The Medical School of Chinese People’s Liberation Army (PLA) General Hospital, Beijing, China; ^2^ Department of Radiology, The First Medical Center, Chinese PLA General Hospital, Beijing, China; ^3^ Department of Radiology, The Sixth Medical Center, Chinese PLA General Hospital, Beijing, China; ^4^ Department of Radiology, Qingdao Special Servicemen Recuperation Center of PLA Navy, Qingdao, China

**Keywords:** diffuse glioma, apparent diffusion coefficient, MRI, isocitrate dehydrogenase status, machine learning, prediction

## Abstract

**Purpose:**

To evaluate isocitrate dehydrogenase (IDH) status in clinically diagnosed grade II~IV glioma patients using the 2016 World Health Organization (WHO) classification based on MRI parameters.

**Materials and Methods:**

One hundred and seventy-six patients with confirmed WHO grade II~IV glioma were retrospectively investigated as the study set, including lower-grade glioma (WHO grade II, n = 64; WHO grade III, n = 38) and glioblastoma (WHO grade IV, n = 74). The minimum apparent diffusion coefficient (ADCmin) in the tumor and the contralateral normal-appearing white matter (ADCn) and the rADC (ADCmin to ADCn ratio) were defined and calculated. Intraclass correlation coefficient (ICC) analysis was carried out to evaluate interobserver and intraobserver agreement for the ADC measurements. Interobserver agreement for the morphologic categories was evaluated by Cohen’s kappa analysis. The nonparametric Kruskal-Wallis test was used to determine whether the ADC measurements and glioma subtypes were related. By univariable analysis, if the differences in a variable were significant (P<0.05) or an image feature had high consistency (ICC >0.8; κ >0.6), then it was chosen as a predictor variable. The performance of the area under the receiver operating characteristic curve (AUC) was evaluated using several machine learning models, including logistic regression, support vector machine, Naive Bayes and Ensemble. Five evaluation indicators were adopted to compare the models. The optimal model was developed as the final model to predict IDH status in 40 patients with glioma as the subsequent test set. DeLong analysis was used to compare significant differences in the AUCs.

**Results:**

In the study set, six measured variables (rADC, age, enhancement, calcification, hemorrhage, and cystic change) were selected for the machine learning model. Logistic regression had better performance than other models. Two predictive models, model 1 (including all predictor variables) and model 2 (excluding calcification), correctly classified IDH status with an AUC of 0.897 and 0.890, respectively. The test set performed equally well in prediction, indicating the effectiveness of the trained classifier. The subgroup analysis revealed that the model predicted IDH status of LGG and GBM with accuracy of 84.3% (AUC = 0.873) and 85.1% (AUC = 0.862) in the study set, and with the accuracy of 70.0% (AUC = 0.762) and 70.0% (AUC = 0.833) in the test set, respectively.

**Conclusion:**

Through the use of machine-learning algorithms, the accurate prediction of IDH-mutant versus IDH-wildtype was achieved for adult diffuse gliomas *via* noninvasive MR imaging characteristics, including ADC values and tumor morphologic features, which are considered widely available in most clinical workstations.

## Introduction

Cerebral diffuse infiltrating gliomas are the second most common type of primary central nervous system (CNS) tumor, second only to meningiomas. According to the 2016 World Health Organization (WHO) classification of CNS tumors, adult diffuse gliomas include astrocytic tumors, oligodendrogliomas, and glioblastomas (WHO grade II~IV) (1). These tumors account for approximately 22% of all CNS tumors. In the United States, more than 16,000 cases of adult diffuse glioma are reported each year, with an incidence of approximately 5.13 per 100,000 people. In addition, glioblastoma (GBM) is the most common malignant tumor in the CNS, accounting for approximately 14.6% of all CNS tumors and 48.3% of all malignant CNS tumors, with 11,833 cases reported annually within the U.S (2, 3). However, due to the heterogeneity of these neuroepithelial tumors, they have different clinical characteristics, biological behaviors, and histopathological characteristics, and substantial differences in treatment and prognosis.

Recently, the isocitrate dehydrogenase (IDH) status and other molecular subtypes have been reported as major prognostic factors and molecular diagnostic criteria for glioma tumor behavior. Thus, noninvasively detecting molecular subtypes before surgery is important for predicting the outcome and choosing the best therapy. Previous studies have shown that lower-grade glioma (LGG) IDH-wildtype and glioblastoma (GBM) have similar molecular structures and prognoses, while IDH-mutant status confers longer overall survival than IDH-wildtype status (4). In addition, compared with glioblastomas in patients with IDH-mutations (grade IV), anaplastic gliomas (grade III) in patients with wild-type IDH have a worse prognosis (5). It should be noted that IDH mutation status has been integrated into the 2016 WHO Classification of Tumors of the Central Nervous System, Revised 4th edition (1). Furthermore, it has been reported that due to different molecular subtypes, the choice of surgical resection range has different survival effects on patients with lower-grade glioma (grades II and III) (6). Based on the above research (5–7), it is necessary to predict the IDH status accurately before surgery and to guide the clinical development of appropriate tumor treatment plans.

Diffusion-weighted imaging (DWI) is a practical imaging technique that is widely employed in the clinic and is mainly used to detect the diffusion of water molecules (8). A meta-analysis showed that the quantitative measurement of the apparent diffusion coefficient (ADC) value can be used to grade gliomas with high accuracy (9). Our previous study demonstrated that the minimum ADC (ADCmin) can be used to predict the grading of neuroepithelial tumors (10). Prior studies (11, 12) have shown that the characteristics of lesions, such as location, internal structure, and enhancement pattern, are different among the genetic subtypes of glioma. In addition, machine learning has been applied in different medical fields, including medical image interpretation, prediction of disease development, and treatment response (13, 14). The advantage of machine learning is that it does not require any assumptions about the input variables and their relationships with the output; in addition, it is a fully data-driven learning method that does not rely on rules-based programming. Therefore, our study focused on the WHO 2016 classification criteria, applying machine learning methods to evaluate the value of clinically obtainable MRI features in predicting the IDH status of adult patients with diffuse grade II~IV glioma.

## Materials and Methods

### Patient Cohort

**Figure 1:**
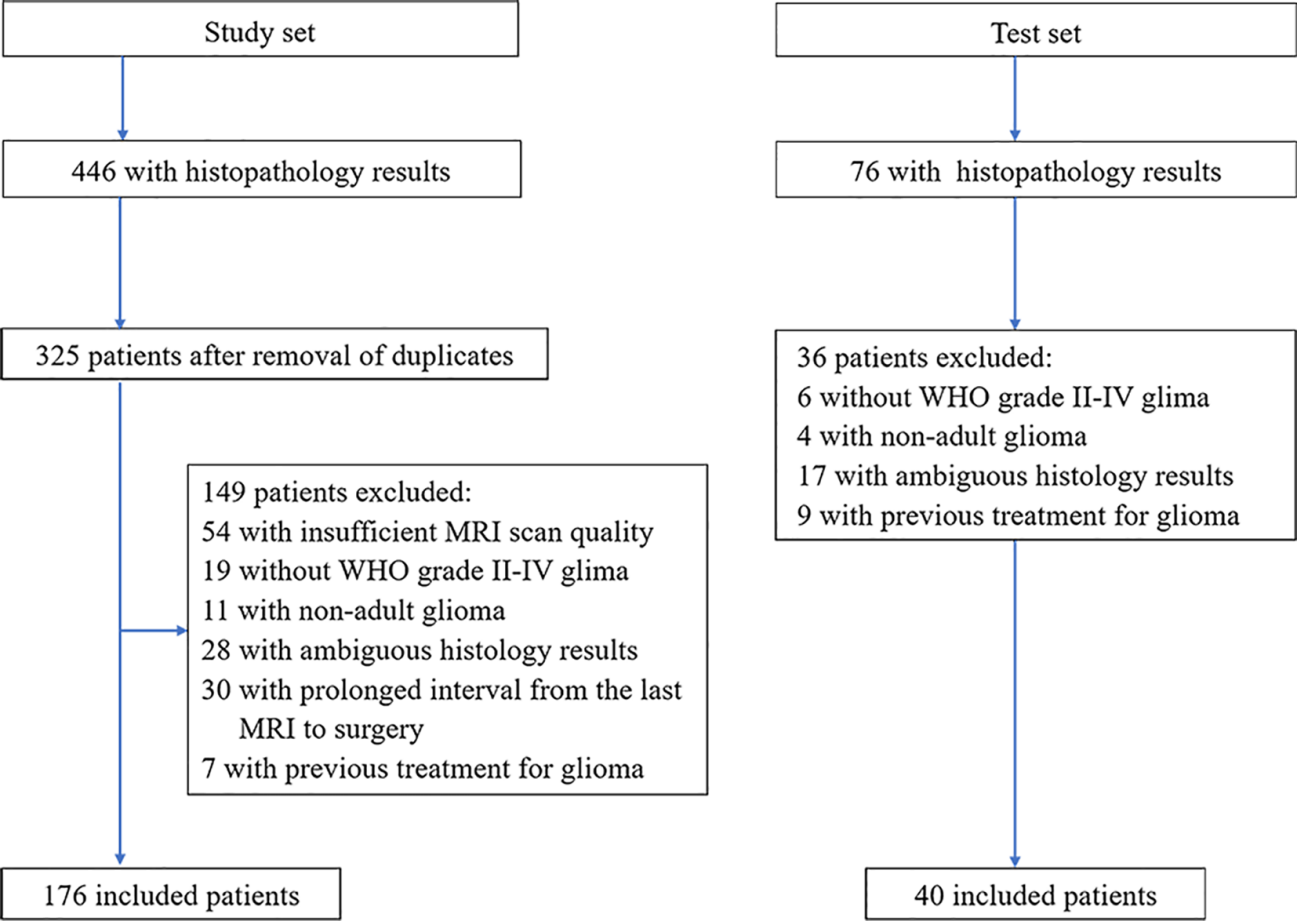
Patient selection flowchart. ADC, apparent diffusion coefficient; WHO, World Health Organization.

This retrospective study was approved by the Institutional Ethics Committee of the Chinese PLA General Hospital, which waived the requirement for written informed consent. From August 2015 to July 2020, through the hospital’s local picture archiving and communication system (PACS), two radiologists (Z.J. and P.H., with 10 and 13 years of experience, respectively), continuously collated patients with WHO grade II~IV glioma who underwent brain MRI. The original study cohort was collected from August 2015 to December 2019 as the study set, and another 40 cases from January 2020 to June 2020 were collected as the test set. The inclusion criteria included (a) a confirmed histologic diagnosis in accordance with WHO grade II-IV glioma; (b) conclusive histopathological and immunohistochemical staining results; and (c) brain MRI examinations performed within 6 months of WHO II/III and within 5 weeks of WHO IV prior to neurosurgical treatment. The exclusion criteria included (a) an MRI scan with substandard quality, including an incomplete MRI protocol, the inability to compute the ADC map and obvious artifacts; (b) tumors other than WHO grade II~IV adult glioma; (c) incomplete or ambiguous histologic results; and d) previous treatment for glioma, such as radiotherapy, chemotherapy or immunotherapy. The flow chart of the enrolled patients (including the study set and test set) is provided in **Figure 1**.

### MRI Examination

All enrolled patients underwent 3.0 T MRI. The MRI protocols included axial T2-weighted, axial or coronal T2 FLAIR, axial T1-weighted, fat-suppressed contrast-enhanced T1-weighted (including axial, coronal and sagittal) imaging, susceptibility-weighted imaging (SWI) and diffusion-weighted imaging. DWI was performed with b values of 0 s/mm^2^ and 1000 s/mm^2^ and was used to derive the ADC maps. Our institution is a general hospital, and although the MRI scans came from several examination rooms, they were performed with the same system (GE Healthcare, Milwaukee, USA). The MRI machines and protocols used are provided in **Supplementary Table 1**.

### Histopathologic Analysis

All tumors were surgically resected, and the lesion specimens were fixed with paraffin blocks during the operation. Then, the neurologic pathology group adopted the 2016 WHO glioma classification for gross pathology and immunohistochemical staining to analyze and provide the final results.

### ADC Quantification

The interobserver and intraobserver levels of agreement for ADC were assessed from the measurements made by two blinded radiologists (JZ and HP, with 10 and 13 years of experience, respectively, both with professional qualification certificates). To assess intraobserver reproducibility, the first observer performed region of interest (ROI) delineation twice within one week following the same procedure each time. At the same time, the second observer independently delineated the ROI once, and the interobserver agreement was assessed by comparing the results with the ADC outcomes extracted from the first ROI delineation made by the first observer.

Three different ROIs (30-40 mm^2^) were placed into the visually perceived lowest portions inside the tumors on the ADC maps, excluding hemorrhagic, cystic, and necrotic portions and calcifications that might influence the measured results without overlapping the ROIs. Then, the minimum ADC was defined as the average value of the ROIs with the lowest ADC values, as in Maynard et al. (11) and Xing et al. (12). Subsequently, following the same method, an ROI was delineated by selecting the contralateral centrum semiovale region (8, 11), and defining the ADC value within it as ADCn. Thus, there were four ROIs per patient. Finally, the rADC (ADCmin to ADCn ratio) was calculated, resulting in three total ADC parameters (ADC_min_, ADCn, rADC) per patient.

**Figure 2:**
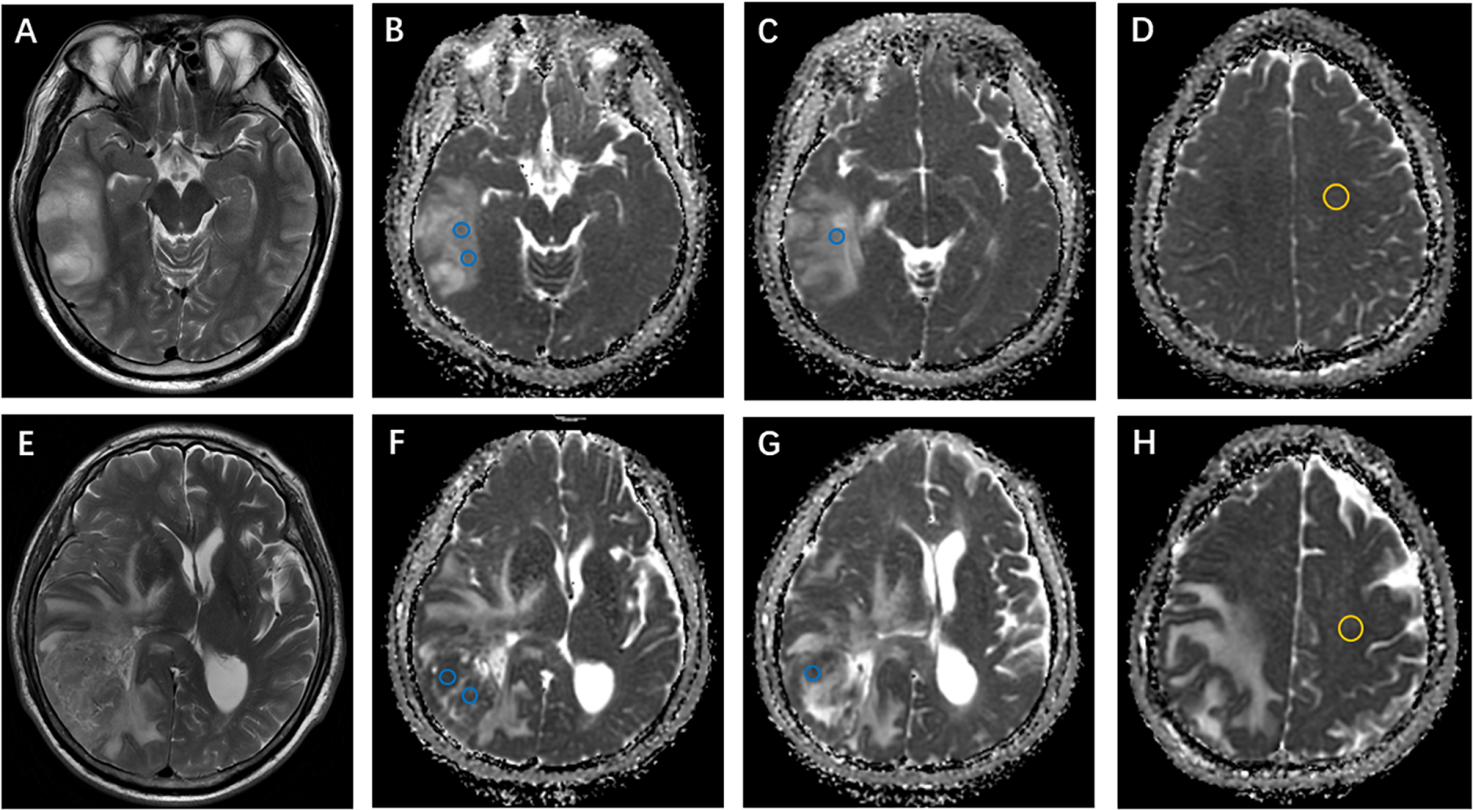
Examples of apparent diffusion coefficient (ADC) measurements. **(A–D)** Axial T2-weighted imaging shows an isocitrate dehydrogenase (IDH)-mutant oligodendroglioma in the right temporal lobe. ADC maps show the regions of interest used to determine ADCmin (perceived lowest ADC regions, blue) and ADCn (contralateral, normal-appearing white matter, yellow). **(E–H)** Axial T2-weighted imaging of a right temporal IDH-wildtype glioblastoma. ADCmin and ADCn were calculated using the same method as above.

In the test set (n = 40), all ADC values were obtained by two certificated radiologists (Y-YC and Y-LW, with 3 and 18 years of experience, respectively) according to the method described above. Examples of ROI delineations are shown in **Figure 2**.

### Morphologic Assessment

Two board-certified radiologists (JZ and HP with 10-13 years of experience) independently evaluated 176 MRI datasets in this study for 1 month while being blinded to the pathologic results.

**Figure 3:**
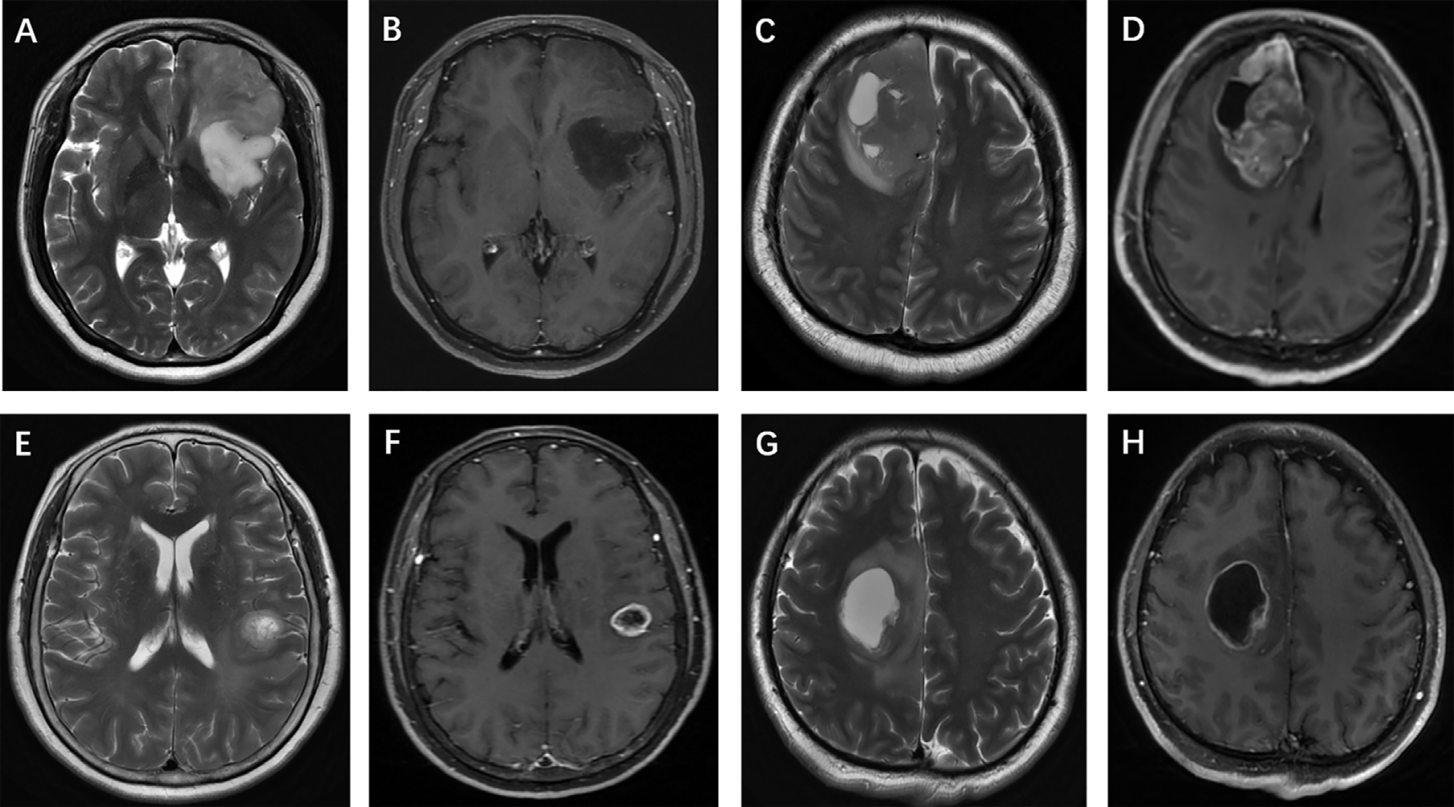
Glioma morphologic characteristics. Enhancement pattern: **(A, B)** T2-weighted imaging shows a left frontal isocitrate dehydrogenase (IDH)-mutant oligodendroglioma without contrast agent uptake; **(C, D)** T2-weighted imaging shows a right frontal IDH-wildtype glioblastoma, and contrast-enhanced T1-weighted imaging shows patchy contrast uptake; **(E, F)** T2-weighted imaging and contrast-enhanced T1-weighted imaging show rim enhancement surrounding a central necrosis in a IDH-wildtype astrocytoma, while another patient **(G, H)** presents with a frontal IDH-mutant glioblastoma.

**Figure 4:**
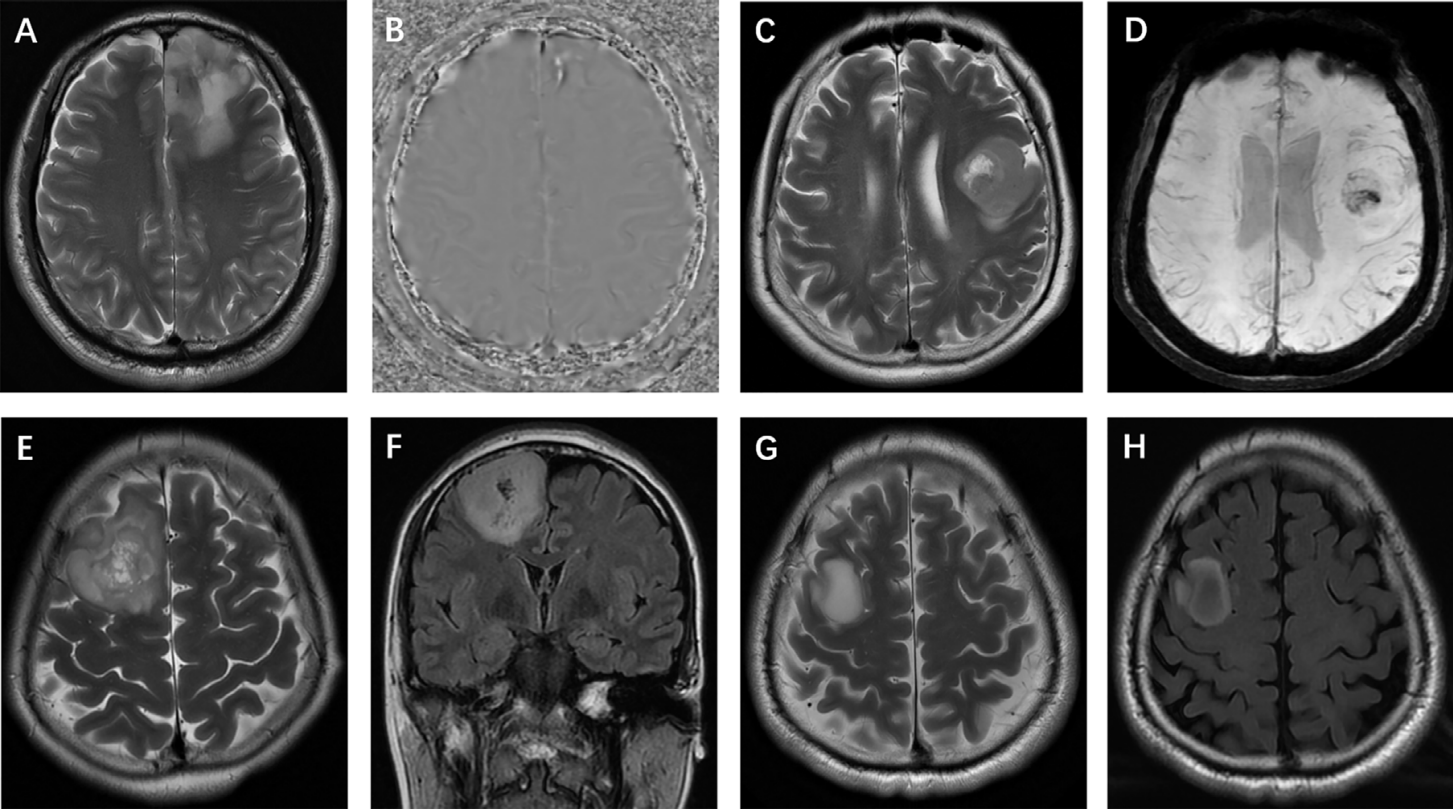
Glioma morphologic characteristics. **(A, B)** Calcification: T2-weighted imaging and phase image on SWI show calcification in a left frontal isocitrate dehydrogenase (IDH)-mutant oligodendroglioma; **(C, D)** Hemorrhage: T2-weighted imaging and SWI show hemorrhage in a left frontal IDH-wildtype glioma; **(E, F)** Cystic change: T2-weighted and FLAIR imaging show small cysts in a mutant-IDH oligodendroglioma; **(G, H)** T2-FLAIR mismatch sign: T2-weighted and FLAIR images show a T2-FLAIR mismatch sign in a mutant-IDH diffuse astrocytoma.

The selection and evaluation of the tumor morphology were performed according to previous publications (11, 12). (a) Tumor location, which was specified by the center of the lesion, was divided into 4 groups: frontal lobe, other lobes (including parietal lobe, temporal lobe and occipital lobe), thalamus or brainstem, and cerebellum. (b) The maximum tumor diameter was measured by reference to the T2-weighted images, FLAIR images and contrast-enhanced T1-weighted images. (c) Contrast enhancement was categorized into 3 groups: nonenhancement, patchy enhancement, and rim enhancement. (d) Calcification and hemorrhage were observed and evaluated on T1-weighted imaging, susceptibility-weighted imaging, and CT, as available. (e) Cystic changes and central necrosis were defined as a free-liquid intensity with a nonenhanced portion. (f) T2-FLAIR mismatch signs, which previous studies considered to be specific (15, 16), were defined as tumors showing nearly homogeneous hyperintensity on T2-weighted images and relatively low intensity and peripheral hyperintensity on FLAIR sequences. **Figures 3** and **4** show examples of different morphologic characteristics of gliomas on MRI in the study set.

### Statistical Analysis

Statistical analyses were performed using SPSS (version 26.0) and Python (version 3.8). Intraclass correlation coefficient analysis was used to evaluate the interobserver and intraobserver levels of agreement for ADC measurements, applying a two-way random-effects model. The interobserver agreement for morphologic categories was evaluated by Cohen’s kappa analysis. For the agreement analysis, the outcomes were interpreted as follows: 0.2 or less, slight agreement; 0.21–0.40, fair agreement; 0.41–0.60, moderate agreement; 0.61–0.80, substantial agreement; and 0.81–1.00, almost perfect agreement.

The differences in ADC values among IDH subtype glioma groups were tested using nonparametric Kruskal-Wallis test. The relationship between morphologic features and glioma subtypes was analyzed using the chi-squared test. P<0.05 was considered to indicate a statistically significant difference.

In the univariable analysis, if the differences in a variable were significant (P<0.05) or an image feature had high consistency (ICC >0.8; k >0.6), then it was chosen as a predictor variable for multivariable logistic regression to predict IDH subtypes of glioma.

### Model Construction

For machine learning, we attempted to implement the following machine learning methods, which are currently the most popular machine learning methods used to classify glioma tumors (17–20), to develop prediction models: logistic regression, support vector machine (SVM), Naive Bayes (NB) and Ensemble (random forest + eXtreme Gradient Boosting). The logistic regression model uses the maximum likelihood method to estimate and determine the regression coefficient and accurately predict the probability of dichotomy. SVM is a supervised learning algorithm that can clearly identify high-dimensional boundaries and solve dichotomy problems (21). Ensemble algorithms include random forest and eXtreme Gradient Boosting. Random forest is an integrated algorithm that combines multiple decision trees together by voting to discriminate and classify data (22). eXtreme Gradient Boosting integrates many weak classifiers into a strong classifier, which is an optimized extreme gradient promotion to improve the predictive power (21, 23). We also attempted NB, an efficient algorithm based on the Bayesian principle that uses the knowledge of probability in statistics to classify data sets (24). The construction process for each model is provided in **Supplementary Data**.

To evaluate the predictive accuracy of these machine learning models and select the most suitable model, we calculated and compared sensitivity, specificity, accuracy, the areas under the receiver operating characteristic curve (AUC) and F1 score (25). Then, the best machine learning model was chosen as the final model to evaluate the IDH subtype probability in the test set. In clinical practice, SWI and CT, which help to observe calcification, may be unavailable in some circumstances, an alternative model (model 2) was developed in which calcification status was excluded from the multivariable logistic regression model. Subgroup analysis was also performed to validate the final model on LGG and GBM. DeLong analysis was used to compare significant differences in the AUCs (26).

## Results

### Patients Demographic Characteristics

The flow chart of the enrolled patients (including the study and test sets) is provided in **Figure 1**. After excluding patients because of non-adult patients (age<18 y, n=11), insufficient MRI scan quality (n=54), the presence of tumors other than WHO grade II-IV glioma (n=19, including 8 WHO grade I and 11 diffuse midline gliomas), ambiguous histology results (n=28), a duration from MRI to surgery longer than 6 months in WHO II/III or 5 weeks in WHO IV (n=30), or a previous treatment for glioma (n=7). A total of 176 patients (109 male and 67 female patients; mean age, 46.5 years; age range, 21-74 years) with lower-grade glioma (n=102) and glioblastoma (n=74) were ultimately enrolled in the analysis of the study set. There was no relationship found between glioma IDH subtype and sex, but patients with the IDH-wildtype status were more likely to be older than those with the IDH-mutant status, especially in cases of GBM. An overview of patient information, morphologic features and IDH subgroups is listed in **Table 1** and **Supplemental Table 2**.

**Table 1:** Patient demographics and MRI morphological characteristics in the study set.

Parameter	All Gliomas	IDH Mutation	IDH Wild-Type	P value
**Number of Patients**	176	89	87	
**Age**	46.5 [35.0,54.0]	41.0 [33.0,49.0]	50.0 [40.0,59.0]	<0.001
**Sex**				
**Female**	67 (38.1)	31 (34.8)	36 (41.4)	0.460
**Male**	109 (61.9)	58 (65.2)	51 (58.6)	
**Tumor Location**				
**Frontal lobe**	89 (50.6)	56 (62.9)	33 (37.9)	0.010
**Other lobes**	74 (42.0)	29 (32.6)	45 (51.7)	
**Thalamus or brainstem**	9 (5.1)	3 (3.4)	6 (6.9)	
**Cerebellum**	4 (2.3)	1 (1.1)	3 (3.4)	
**Diameter**				
**<6 cm**	132 (75.0)	62 (69.7)	70 (80.5)	0.139
**≥6 cm**	44 (25.0)	27 (30.3)	17 (19.5)	
**Enhancement**				
**Nonenhancement**	63 (35.8)	53 (59.6)	10 (11.5)	<0.001
**Patchy enhancement**	42 (23.9)	25 (28.1)	17 (19.5)	
**Ring enhancement**	71 (40.3)	11 (12.4)	60 (69.0)	
**Calcification**				
**No**	152 (86.4)	68 (76.4)	84 (96.6)	<0.001
**Yes**	24 (13.6)	21 (23.6)	3 (3.4)	
**Cystic Change**				
**No**	73 (41.5)	48 (53.9)	25 (28.7)	0.001
**Yes**	103 (58.5)	41 (46.1)	62 (71.3)	
**Hemorrhage**				
**No**	91 (51.7)	63 (70.8)	28 (32.2)	<0.001
**Yes**	85 (48.3)	26 (29.2)	59 (67.8)	
**T2 FLAIR Mismatch**				
**No**	136 (77.3)	62 (69.7)	74 (85.1)	0.024
**Yes**	40 (22.7)	27 (30.3)	13 (14.9)	
**WHO 2016 Grade**				
**Lower-grade Glioma**	102 (58.0)	78 (88.7)	23 (26.4)	<0.001
**Glioblastoma**	74 (42.0)	10 (11.2)	64 (73.6)	

Data in parentheses are ranges, and data in brackets are interquartile ranges.

IDH, isocitrate dehydrogenase; FLAIR, fluid-attenuated inversion recovery.

### Morphologic Assessment Results

Regarding tumor location, the measured values demonstrated almost perfect interobserver agreement (κ=0.835, P<0.01). For the longest tumor diameter (<6 cm or ≥6 cm), the measurement reached almost perfect agreement (κ=0.848, P<0.01). The determination of calcification showed substantial agreement (κ=0.719, P<0.01). Determination of the presence of a cyst or necrosis reached almost perfect agreement (κ=0.862, P<0.01). For the enhancement patterns, the results demonstrated substantial agreement (κ=0.786, P<0.01). The determination of hemorrhage reached almost perfect agreement (κ=0.852, P<0.01). In assessing the T2-weighted FLAIR mismatch sign, fair interobserver agreement was found (κ=0.396, P<0.01). Cohen’s kappa results for the morphology categories are provided in **Supplementary Table 3**.

### ADC Quantification

**Figure 5:**
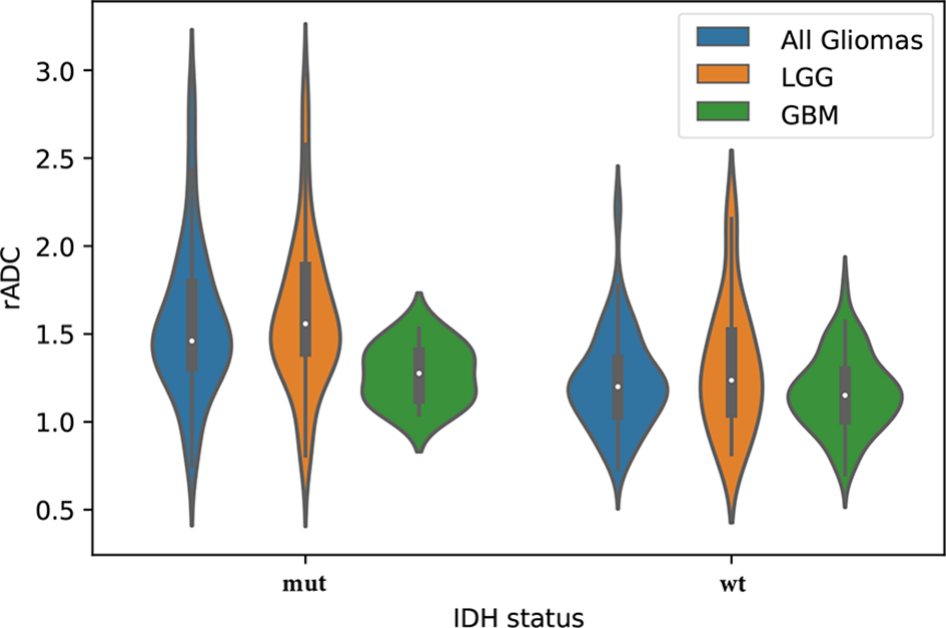
The violin plot shows differences in the apparent diffusion coefficient (ADC) values (ADCmin to ADCn ratio, rADC) between the isocitrate dehydrogenase (IDH) statuses (mut = IDH-mutant, wt = IDH-wildtype) in the study set, including lower-grade glioma (LGG) and glioblastoma (GBM).

The interobserver and intraobserver levels of reproducibility were almost perfect for all ADC parameters (ICC=0.80-0.95), which indicated that there was no systematic difference between the observers. The rADC correctly classified IDH-mutant and IDH-wildtype in WHO grade II~IV gliomas and LGG subgroup (P<0.05), but not in GBM subgroup (P=0.126). The results are shown in **Figure 5**. Nonparametric testing (Kruskal-Wallis analysis of variance) revealed an association between ADC value and IDH status (P<0.001). The ICCs for different ADC values are provided in **Supplementary Tables 4 **and** 5**.

### Predictor Selection (Univariable Analysis and Machine Learning Model)

The chi-squared tests revealed associations between morphological features, including enhancement, calcification, cysts, hemorrhage, cystic change and T2-FLAIR mismatch, and IDH status (P<0.05). The univariable analysis results are shown in **Table 2**.

**Table 2:** Crude association between IDH status and ADC value and morphologic features for the study set.

	Values	OR	P	95% CI
**rADC**	1.39 ± 0.39	0.05	<0.001	(0.01, 0.15)
**ADCmin**	1.02 ± 0.27	0.01	<0.001	(0.00, 0.07)
**Age**	45.30 ± 12.53	1.05	0.001	(1.02, 1.08)
**Sex**				
**Female**	67 (38.07%)	Reference		
**Male**	109 (61.93%)	0.76	0.372	(0.41, 1.39)
**Tumor Location**				
**Frontal lobe**	89 (50.57%)	Reference		
**Other lobes**	74 (42.05%)	2.63	0.003	(1.40, 4.97)
**Thalamus or brainstem**	9 (5.11%)	3.39	0.100	(0.80, 14.49)
**Cerebellum**	4 (2.27%)	5.09	0.166	(0.51, 50.97)
**Diameter**				
**<6 cm**	132 (75.00%)	Reference		
**≥6 cm**	44 (25.00%)	0.56	0.100	(0.28, 1.12)
**Enhancement**				
**Nonenhancement**	63 (35.80%)	Reference		
**Patchy enhancement**	42 (23.86%)	3.6	0.006	(1.44, 8.99)
**Ring enhancement**	71 (40.34%)	28.91	<0.001	(11.38,73.47)
**Calcification**				
**No**	152 (86.36%)	Reference		
**Yes**	24 (13.64%)	0.12	0.007	(0.03, 0.40)
**Cystic Change**				
**No**	73 (41.48%)	Reference		
**Yes**	103 (58.52%)	2.9	0.008	(1.56, 5.42)
**Hemorrhage**				
**No**	91 (51.70%)	Reference		
**Yes**	85 (48.30%)	5.11	<0.001	(2.69, 9.69)
**T2-FLAIR Mismatch**				
**No**	136 (77.27%)	Reference		
**Yes**	40 (22.73%)	0.4	0.017	(0.19, 0.85)

**Figure 6:**
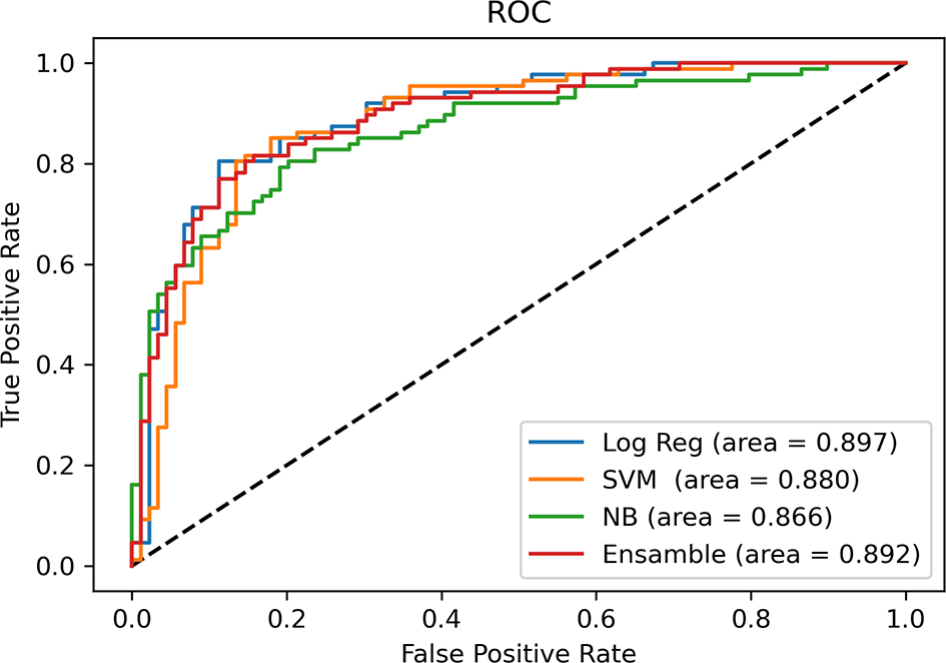
Comparison of AUCs among machine learning models. Receiver operating characteristic curves are shown for logistic regression (Log Reg), support vector machine (SVM), Naive Bayes (NB) and Ensemble (random forest + eXtreme Gradient Boosting) in predicting the IDH status of glioma.

**Figure 7:**
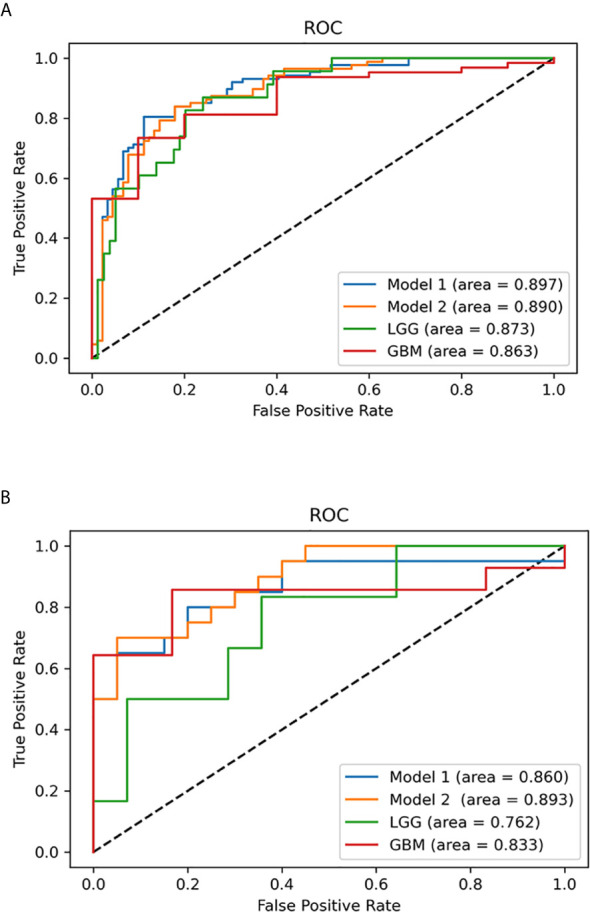
Multivariable logistic regression analysis was used to predict isocitrate dehydrogenase (IDH) status in the study set and test set. **(A)** Receiver operating characteristic (ROC) curves of the multivariable probabilities for models 1 and 2, lower-grade glioma (LGG) and glioblastoma (GBM) in the study set. **(B)** ROC curves of the multivariable probabilities for model 1, model 2, LGG and GBM in the test set.

After univariable analysis selection, combined with features with substantial agreement (k >0.6), six measured variables were selected for incorporation into the machine learning model, including rADC, age, enhancement, calcification, hemorrhage, and cystic change. In terms of the prediction accuracy of the single model, logistic regression, SVM, NB and ensemble showed similar model performance to the study set (AUC=0.866-0.897). Among them, logistic regression exhibited the largest area under the curve (AUC= 0.897) and the model achieved better performance than others. Then, we chose multivariable logistic regression as the final model. Models 1 and 2 (not including calcification) performed almost equivalently, with an AUC of 0.890 for model 2. DeLong analysis showed no statistically significant difference between the two models (P=0.361). In the lower-grade glioma and GBM, the models also achieved better performance, with the accuracy of 84.3% (AUC = 0.873) and 85.1% (AUC = 0.862), respectively. The AUCs of the different machine learning models are presented in **Figure 6**. The comparison of machine learning models is provided in **Supplementary Table 6**. The results of models 1 and 2, LGG and GBM are shown in **Table 3** and **Figure 7**.

**Table 3:** Multivariable logistic regression results for predicting the IDH status in the study set and the test set.

	Number	Se	Sp	PPV	NPV	YI	Accuracy	AUC
**Study set**								
**Model 1**	176	0.805	0.888	0.856	0.816	0.693	0.824	0.897
**Model 2**	176	0.839	0.820	0.823	0.836	0.659	0.812	0.890
**LGG**	102	0.870	0.759	0.783	0.854	0.629	0.843	0.873
**GBM**	74	0.734	0.900	0.880	0.772	0.634	0.851	0.862
**Test set**								
**Model 1**	40	0.800	0.800	0.800	0.800	0.600	0.750	0.860
**Model 2**	40	0.700	0.950	0.933	0.760	0.650	0.750	0.893
**LGG**	20	0.833	0.643	0.700	0.794	0.476	0.700	0.762
**GBM**	20	0.857	0.833	0.837	0.853	0.690	0.700	0.833

Se, sensitivity; Sp, specificity; PPV, positive predictive value; NPV, negative predictive value; YI, Youden Index; AUC, area under the curve; LGG, lower-grade glioma; GBM, glioblastoma.

### Test Set Results

To predict the probability of the IDH status of patients in the subsequent test set, the study set results were transcribed into Python for further calculation. From January 2020 to June 2020, 40 diagnosed glioma patients (20 with IDH-mutant and 20 with IDH-wildtype) were included in the test set according to the same inclusion criteria. Two blinded observers (Y-YC and Y-LW) replicated the ADC measurements used in the study set. The ICCs for different ADC values are provided in **Supplementary Table 4**. The AUCs of models 1 and 2, LGG and GBM in the test set are presented in **Table 3** and **Figure 7**. Model 1 correctly classified the IDH status in the test set (AUC=0.860), with sensitivity of 80% and specificity of 80%. Model 2 performed well in predicting the IDH status of glioma (AUC=0.893), demonstrating a greater specificity of 95% but a lower sensitivity of 70% than model 1. Subgroup analysis revealed that the model predicted IDH status of LGG and GBM with the accuracy of 70.0% (AUC = 0.762) and 70.0% (AUC = 0.833), respectively.

## Discussion

In this study, machine learning methods were developed and validated, combining rADCs with tumor morphologic characteristics to predict the IDH status of adult WHO grade II-IV gliomas. In the predictive models, the logistic regression model exhibited the greatest AUC (0.897). Two models, model 1 (including age, rADC, enhancement pattern, calcification, cystic change and hemorrhage) and model 2 (excluding calcification), were developed and correctly classified the IDH status with similar model performance for the study set (n=176, AUC=0.890-0.897) and a previously unseen test set (n = 40, AUC=0.860-0.893).

Previous studies have analyzed the association between MRI features and the IDH status of lower-grade gliomas (WHO grade II-III) and glioblastomas (WHO grade IV) [specifically, Thust et al. and Xing et al. evaluated the features of grade II/III gliomas (8, 12), while Zhang et al. identified MRI features associated with grade III and IV gliomas (7)]. To our knowledge, no previous attempts have been made to use different machine learning methods to build a suitable model combining clinical and magnetic resonance imaging features to predict the IDH molecular subtype for WHO grade II to IV gliomas. Furthermore, previous studies have used region-derived minimum ADC measurements to estimate glioma grade or molecular status (8, 11, 12, 27, 28). Not surprisingly, according to receiver operating characteristic curve analysis, the ADC value was shown to be a useful tool for detecting the IDH status in diffuse gliomas, and we found that there was a significant difference between IDH-mutant and IDH-wildtype gliomas (P<0.001). Our study revealed excellent interobserver and intra-observer reproducibility (ICC=0.80-0.95) for ROI measurements, similar to the repeatability results for ADC measurements described in other studies (8). The rADC (ADCmin to nADC ratio) was used as a fixed parameter to ensure vendor neutrality and to reduce the potential bias. When drawing the ROI, this study only included the solid part, avoiding cystic or necrotic portions and hemorrhagic areas as much as possible, which is considered feasible on most clinical workstations. This method is partially consistent with the results reported by G.Z (29) who suggested that when drawing ROIs on ADC maps, selection of the solid part is necessary and is an optimal choice for differentiating GBM from metastasis.

When testing the rADC for predicting IDH status, our study found that the ADCmin and rADC of IDH-mutant glioma were higher than those of IDH-wildtype glioma in WHO grade II~IV gliomas and LGG subgroup, but not in GBM subgroup. ADCmin has been confirmed to represent the area with the highest cellularity in heterogeneous tumors. In general, the lower the ADC value is, the denser the glioma cells, and the worse the prognosis, which is supported by several studies comparing diffusivity, histological specimens and clinical data (8, 30). Hong et al. reported that ADC was significantly lower in IDH-wildtype GBM than in IDH-mutant GBM (31). However, our study failed to find this result. One reason may be attributed to the difference in sample size, with only 10 IDH mutants in our GBM subgroup. The other reason may be due to the heterogeneity in GBM and different ROI biases. Glioblastomas have different subsets of genetic abnormalities that take part in tumorigenesis and transformation, especially IDH mutants, which may contain lower-grade tumor components (32). In our study, the lowest value of ADC was selected for analysis, which greatly avoided the measurement bias caused by measuring the whole tumor.

Although quantitative, computerized methods hold substantial promise for the noninvasive prediction of the molecular characteristics of glioma, we aimed to establish a model by combining several morphologic features that can be easily evaluated on conventional, standard MRI daily in the clinic. Considering the age and morphological characteristics of our population, consistent with previous research, younger age and forehead positions were more likely to be associated with mutation status (33, 34). Arita et al. (35) found that IDH-wildtype gliomas were mainly distributed in the parietal lobe and, to some extent, the temporal lobe but were rarely involved the frontal lobe. In our study, IDH-wildtype status was similarly associated with a greater likelihood of distribution in cerebral lobes other than the frontal lobe. Moreover, thalamic or brainstem locations and cerebellar locations showed IDH-wildtype predominance, which concurs with a study by Maynard et al. (11).

Our study showed a significant difference in postcontrast enhancement patterns between glioma subtypes in WHO grade II-IV glioma. Indeed, tumor ring-enhancement is a predictor of IDH-wildtype status, indicating a tendency for invasive behavior. While it is increasingly recognized that nonenhancement tumors also comprise a substantial proportion of grade IV gliomas (36), it should be noted that images of atypical glioblastoma might not be easily distinguished from lower-grade gliomas on routine MRI. Furthermore, the presence of hemorrhage was not related to a particular subgroup in our study. Moreover, previous studies show that the T2-FLAIR mismatch sign has high specificity in diagnosing IDH-mutant astrocytoma (16). This tendency was also shown in our research results, but it was not selected for incorporation into the model due to the fair interobserver agreement (κ=0.396).

Calcification and cystic components also significantly contributed to our predictive model in WHO grade II-IV glioma. The absence of calcification strongly correlated with the IDH-wildtype status in univariable analysis. This finding is consistent with previous studies that have extensively evaluated calcification in IDH-mutant gliomas (11). The interobserver agreement was moderate (κ=0.719, P<0.01). We hypothesize that by expanding the sample size and optimizing the examination sequence, the certainty and concordance of the observers would further increase when observing calcification. Kanazawa et al. (37) found that both calcification and cystic components could be used to predict IDH-mutant status with 1p/19q deletion in lower-grade gliomas. However, in our study, cystic components were more likely to be found in IDH-wildtype tumors than in IDH-mutant tumors. Considering that IDH-wildtype tumors are more necrotic than IDH-mutant tumors (38), we speculate that subjectivity and overlap with necrotic components limit the reproducibility of this correlation.

Several limitations of the current study should be noted. First, we did not include infantile gliomas because high-grade gliomas are a specific entity with a paradoxical clinical course that distinguishes them from their pediatric and adult counterparts (39). Second, the simplified description and measurements of the ADC values combined with DWI cannot fully reflect the complexity of cell components and structural changes; a more advanced MRI postprocessing method (for example, a method that uses semiautomatic or automatic segmentation to cover the total tumor volume) may partially overcome these limitations at the expense of more time-consuming preprocessing and postprocessing workflows. It is worth mentioning that our ADC measurements applied are available in most clinical workstations. Finally, our study is a retrospective study based on data from a single institution. The stability of the morphological features may be affected by differences in the MR parameters and protocol, the image postprocessing steps and the repeatability of ADC measurements. Therefore, the next step is to conduct a multi-center study to verify our inferences.

In conclusion, we demonstrated that the ADCmin to ADCn ratio, combined with tumor morphologic features, has high accuracy in predicting tumors with IDH-mutant status versus tumors with IDH-wildtype status in adult diffuse glioma. The combination may provide a noninvasive, significant and feasible alternative marker. Further studies in larger sample trials are needed to improve its clinical application value.

## Data Availability Statement

The original contributions presented in the study are included in the article/**Supplementary Material**. Further inquiries can be directed to the corresponding author.

## Ethics Statement

The studies involving human participants were reviewed and approved by The Institutional Ethical Committee of the Chinese PLA General Hospital. Written informed consent for participation was not required for this study in accordance with the national legislation and the institutional requirements.

## Author Contributions

JZ and LM conceived the study design. JZ, HP, Y-YC, X-BB and D-KZ were responsible for patient recruitment and acquired clinical information. JZ, Y-LW and H-FX conducted the quality assurance of image quality. JZ and HP were responsible for statistical analysis. JZ wrote the first draft of this manuscript. Y-LW and LM reviewed the manuscript. All authors contributed to the article and approved the submitted version.

## Conflict of Interest

The authors declare that the research was conducted in the absence of any commercial or financial relationships that could be construed as a potential conflict of interest.
