# Supplementary material for: Predictive Role of the Apparent Diffusion Coefficient and MRI Morphologic Features on IDH Status in Patients With Diffuse Glioma: A Retrospective Cross-Sectional Study
Source: Front Oncol. 2021 May 13;11:640738. doi: 10.3389/fonc.2021.640738 (PMC8155475; doi:10.3389/fonc.2021.640738)
Supplement: Supplementary file 1 [file DataSheet_1.pdf]

Supplementary Data. The construction for each model by Python 3.8.

```
data_train = pd.read_csv('Model 1 in the study set.csv')
data_test = pd.read_csv('Model 1 in the test set.csv')

features = ['rADC', 'Age', 'Enhancement', 'Calcification', 'Cystic_Change', 'Hemorrhage']
data_train[features]= data_train[features].fillna(data_train[features].mean())
train_X = data_train[features]
test_X = data_test[features]
train_Y = data_train['IDH status']
test_Y = data_test['IDH status']
for feature in features:
    mean, std = train_X[feature].mean(), train_X[feature].std()
    train_X.loc[:, feature] = (train_X[feature] - mean) / std
    test_X.loc[:, feature] = (test_X[feature] - mean) / std

# Logistic regression
modelLR = LogisticRegression()
modelLR.fit(train_X, train_Y)
y_pred_prob_lr = modelLR.predict_proba(test_X)[:,1]
fpr_lr, tpr_lr, thresholds_lr = roc_curve(test_Y, y_pred_prob_lr)
roc_auc_lr = auc(fpr_lr, tpr_lr)
precision_lr, recall_lr, th_lr = precision_recall_curve(test_Y, y_pred_prob_lr)
predicted_lr=modelLR.predict(test_X)
optimal_idx = np.argmax(tpr_lr - fpr_lr)
optimal_threshold = thresholds_lr[optimal_idx]
sensitivity = tpr_lr[optimal_idx]
specificity = 1 - fpr_lr[optimal_idx]
accuracy = accuracy_score(test_Y, predicted_lr)
F1 = f1_score(test_Y, predicted_lr, average='macro')
result_LR = []
result_LR = {'model_name': 'LR', 'AUC': round(roc_auc_lr,3), 'Sensitivity': round(sensitivity,3),
 'Specificity': round(specificity,3), 'Accuracy': round(accuracy,3), 'F1': round(F1,3)}
print(result_LR)

# SVM with linear
modelSVMlinear=svm.SVC(kernel='linear', probability=True)
modelSVMlinear.fit(train_X, train_Y)
y_pred_prob_SVMlinear = modelSVMlinear.predict_proba(test_X)[:,1]
fpr_SVMlinear, tpr_SVMlinear, thresholds_SVMlinear = roc_curve(test_Y, y_pred_prob_SVMlinear)
roc_auc_SVMlinear = auc(fpr_SVMlinear, tpr_SVMlinear)
precision_SVMlinear, recall_SVMlinear, th_SVMlinear = precision_recall_curve(test_Y,
y_pred_prob_SVMlinear)
predicted_SVMlinear=modelSVMlinear.predict(test_X)
```

```
optimal_idx = np.argmax(tpr_SVMlinear - fpr_SVMlinear)
optimal_threshold = thresholds_SVMlinear[optimal_idx]
sensitivity = tpr_SVMlinear[optimal_idx]
specificity = 1 - fpr_SVMlinear[optimal_idx]
accuracy = accuracy_score(test_Y, predicted_SVMlinear)
F1 = f1_score(test_Y, predicted_SVMlinear, average='macro')
result_SVMlinear = []
result_SVMlinear = {'model_name': 'SVMlinear', 'AUC': round(roc_auc_SVMlinear,3), 'Sensitivity': round(sensitivity,3),
'Specificity': round(specificity,3), 'Accuracy': round(accuracy,3), 'F1': round(F1,3)}
print(result_SVMlinear)

# Naive Bayes
modelNB= GaussianNB()
modelNB.fit(train_X, train_Y)
y_pred_prob_nb = modelNB.predict_proba(test_X)[:,1]
fpr_nb, tpr_nb, thresholds_nb = roc_curve(test_Y, y_pred_prob_nb)
roc_auc_nb = auc(fpr_nb, tpr_nb)
precision_nb, recall_nb, th_nb = precision_recall_curve(test_Y, y_pred_prob_nb)
predicted_NB=modelNB.predict(test_X)
optimal_idx = np.argmax(tpr_nb - fpr_nb)
optimal_threshold = thresholds_nb[optimal_idx]
sensitivity = tpr_nb[optimal_idx]
specificity = 1 - fpr_nb[optimal_idx]
accuracy = accuracy_score(test_Y, predicted_NB)
F1 = f1_score(test_Y, predicted_NB, average='macro')
result_NB = []
result_NB = {'model_name': 'NB', 'AUC': round(roc_auc_nb,3), 'Sensitivity': round(sensitivity,3),
  'Specificity': round(specificity,3), 'Accuracy': round(accuracy,3), 'F1': round(F1,3)}
print(result_NB)

# Ensamble
linear_svm=svm.SVC(kernel='linear', C=0.1, gamma=10, probability=True)
radial_svm=svm.SVC(kernel='rbf', C=0.1, gamma=10, probability=True)
lr = LogisticRegression(C=0.1)
ensembleModel=VotingClassifier(estimators=[('Linear_svm',linear_svm), ('Radial_svm', radial_svm),
('Logistic Regression', lr)], voting='soft', weights=[2,1,3])
ensembleModel.fit(train_X, train_Y)
y_pred_prob_en = ensembleModel.predict_proba(test_X)[:,1]
fpr_en, tpr_en, thresholds_en = roc_curve(test_Y, y_pred_prob_en)
roc_auc_en = auc(fpr_en, tpr_en)
precision_en, recall_en, th_en = precision_recall_curve(test_Y, y_pred_prob_en)
predicted_en=ensembleModel.predict(test_X)
optimal_idx = np.argmax(tpr_en - fpr_en)
```

```
optimal_threshold = thresholds_en[optimal_idx]
sensitivity = tpr_en[optimal_idx]
specificity = 1 - fpr_en[optimal_idx]
accuracy = accuracy_score(test_Y, predicted_en)
F1 = f1_score(test_Y, predicted_en, average='macro')
result_en = []
result_en = {'model_name': 'en', 'AUC': round(roc_auc_en,3), 'Sensitivity': round(sensitivity,3),
'Specificity': round(specificity,3), 'Accuracy': round(accuracy,3), 'F1': round(F1,3)}
print(result_en)
```
