# Supplementary material for: Predictive Role of the Apparent Diffusion Coefficient and MRI Morphologic Features on IDH Status in Patients With Diffuse Glioma: A Retrospective Cross-Sectional Study
Source: Front Oncol. 2021 May 13;11:640738. doi: 10.3389/fonc.2021.640738 (PMC8155475; doi:10.3389/fonc.2021.640738)
Supplement: Supplementary file 2 [file DataSheet_2.docx]

Supplementary Material

# Supplementary Tables

**Supplementary** **Table 1.** Brain MRI protocols for GE MRI Scanners.

| **Sequence** | **FOV** | **TR** | **TE** | **Bandwidth** | **Slice thickness** | **Slice spacing** |
| --- | --- | --- | --- | --- | --- | --- |
|  |  | **(msec)** | **(msec)** | **(kHz)** | **(mm)** | **(mm)** |
| **Axial T2 PROPELLER** | 24 | 5642 | 93 | 83.3 | 5.5 | 1.5 |
| **Axial T1 FLAIR** | 24 | 1750 | 24 | 41.67 | 5.5 | 1.5 |
| **Axial T2 FLAIR** | 24 | 8506 | 162 | 41.67 | 5.5 | 1.5 |
| **Coronal T2 FLAIR** | 24 | 8527 | 162 | 41.67 | 5.5 | 1.5 |
| **Axial DWI ASSET** | 24 | 3000 | 67.6 | 250 | 6 | 1.5 |
| **Axial CE-T1** | 24 | 1750 | 24 | 41.67 | 5.5 | 1.5 |
| **Coronal CE-T1** | 24 | 1750 | 24 | 62.5 | 5.5 | 1.5 |
| **Sagittal CE-T1** | 24 | 1750 | 24 | 62.5 | 5.5 | 1.5 |
| **Axial SWAN** | 24 | 37 | 25 | 62.5 | 2 | 1.5 |

DWI was performed using three diffusion gradients with b values of 0 and b = 1000 s/mm^2^.

Abbreviations: TE = echo time, TR =repetition time, FLAIR = fluid-attenuated inversion recovery, DWI = diffusion-weighted imaging, SWI = susceptibility-weighted imaging, CE = contrast-enhanced.

**Supplementary Table 2.** Patient Demographics and MRI morphological characteristics in LGG and GBM.

|  | **LGG** | |  | **GBM** | |
| --- | --- | --- | --- | --- | --- |
| **Parameter** | **IDH-mutant** | **IDH-wildtype** |  | **IDH-mutant** | **IDH-wildtype** |
| **Number** | 79 | 23 |  | 10 | 64 |
| **Age** | 41.0 [35.0,50.0] | 40.0 [32.5,53.5] |  | 37.0 [29.0,44.8] | 52.0 [46.5,60.2] |
| **Sex** |  |  |  |  |  |
| **Female** | 28 (35.4) | 11 (47.8) |  | 3 (30.0) | 25 (39.1) |
| **Male** | 51 (64.6) | 12 (52.2) |  | 7 (70.0) | 39 (60.9) |
| **Tumor location** |  |  |  |  |  |
| **Frontal lobe** | 51 (64.6) | 8 (34.8) |  | 5 (50.0) | 25 (39.1) |
| **Other lobes** | 26 (32.9) | 11 (47.8) |  | 3 (30.0) | 34 (53.1) |
| **Thalamus or brainstem** | 2 (2.5) | 2 (8.7) |  | 1 (10.0) | 4 (6.2) |
| **Cerebellum** | 0 (0) | 2 (8.7) |  | 1 (10.0) | 1 (1.6) |
| **Diameter** |  |  |  |  |  |
| **<6 cm** | 56 (70.9) | 18 (78.3) |  | 6 (60.0) | 52 (81.2) |
| **≥6 cm** | 23 (29.1) | 5 (21.7) |  | 4 (40.0) | 12 (18.8) |
| **Enhancement** |  |  |  |  |  |
| **None** | 53 (67.1) | 10 (11.5) |  | 0 (0) | 10 (11.5) |
| **Patchy** | 20 (25.3) | 9 (39.1) |  | 5 (50.0) | 8 (12.5) |
| **Ring** | 6 (7.6) | 6 (26.1) |  | 5 (50.0) | 54 (84.4) |
| **Calcification** |  |  |  |  |  |
| **No** | 59 (74.7) | 21 (91.3) |  | 59 (74.7) | 21 (91.3) |
| **Yes** | 20 (25.3) | 2 (8.7) |  | 20 (25.3) | 2 (8.7) |
| **Cystic change** |  |  |  |  |  |
| **No** | 46 (58.2) | 17 (73.9) |  | 9 (90.0) | 63 (98.4) |
| **Yes** | 33 (41.8) | 6 (26.1) |  | 1 (10.0) | 1 (1.6) |
| **Hemorrhage** |  |  |  |  |  |
| **No** | 60 (75.9) | 17 (73.9) |  | 3 (30.0) | 11 (17.2) |
| **yes** | 19 (24.1) | 6 (26.1) |  | 7 (70.0) | 53 (82.8) |
| **T2-FLAIR Mismatch** |  |  |  |  |  |
| **No** | 53 (67.1) | 22 (95.7) |  | 9 (90.0) | 52 (81.2) |
| **Yes** | 26 (32.9) | 1 (4.3) |  | 1 (10.0) | 12 (18.8) |

Data in parentheses are ranges, and data in brackets are interquartile ranges.

Abbreviations: LGG =lower-grade glioma，GBM = glioblastoma, IDH = isocitrate dehydrogenase, FLAIR = fluid-attenuated inversion recovery.

**Supplementary Table 3.** Cohen’s kappa results for morphology categories (study set, n=87 wild-type–IDH and 89 mutant-IDH tumors).

| **Features** | **κ** | **Standard Error** | **P value** | **Interpretation of Kappa** |
| --- | --- | --- | --- | --- |
| **Tumor Location** | 0.852 | 0.039 | <0.001 | almost perfect agreement |
| **Diameter** | 0.848 | 0.046 | <0.001 | almost perfect agreement |
| **Enhancement** | 0.786 | 0.039 | <0.001 | substantial agreement |
| **Calcification** | 0.719 | 0.083 | <0.001 | substantial agreement |
| **Cystic Change** | 0.862 | 0.038 | <0.001 | almost perfect agreement |
| **Hemorrhage** | 0.852 | 0.039 | <0.001 | almost perfect agreement |
| **T2-FLAIR Mismatch** | 0.396 | 0.083 | <0.001 | fair agreement |

**Supplementary Table 4.** Intraclass correlation coefficients (ICCs) for ADC region of interest (ROI) measurements in the study set.

| **Method** | **Consistency ICC** | **Absolute Agreement ICC** |
| --- | --- | --- |
|  | **(95% CI)** | **(95% CI)** |
| **ADCmin interobserver** |  |  |
| **Individual measurements** | 0.806 (0.748-0.852) | 0.807 (0.748-0.853) |
| **Average measurements** | 0.893 (0.856-0.920) | 0.893 (0.856-0.921) |
| **ADCmin intraobserver** |  |  |
| **Individual measurements** | 0.903(0.872-0.927) | 0.896(0.854-0.925) |
| **Average measurements** | 0.949(0.931-0.962) | 0.945(0.921-0.961) |
| **ADCn interobserver** |  |  |
| **Individual measurements** | 0.798(0.738-0.846) | 0.798(0.737-0.846) |
| **Average measurements** | 0.888(0.849-0.917) | 0.887(0.848-0.916) |
| **ADCn intraobserver** |  |  |
| **Individual measurements** | 0.814(0.758-0.859) | 0.810(0.752-0.856) |
| **Average measurements** | 0.898(0.862-0.924) | 0.895(0.858-0.923) |
| **rADC interobserver** |  |  |
| **Individual measurements** | 0.802(0.743-0.849) | 0.803(0.744-0.850) |
| **Average measurements** | 0.890(0.852-0.919) | 0.891(0.853-0.919) |
| **rADC intraobserver** |  |  |
| **Individual measurements** | 0.897(0.863-0.922) | 0.893(0.857-0.920) |
| **Average measurements** | 0.946(0.927-0.960) | 0.944(0.923-0.959) |

**Supplementary Table 5.** Intraclass correlation coefficients (ICC) for ADC region of interest (ROI) measurements in the test set.

| **Method** | **Consistency ICC** | **Absolute Agreement ICC** |
| --- | --- | --- |
|  | **(95% CI)** | **(95% CI)** |
| **ADCmin inter-observer** |  |  |
| **Individual measures** | 0.856(0.744-0.921) | 0.787(0.383-0.911) |
| **Average measures** | 0.923(0.853-0.959) | 0.881(0.554-0.953) |
| **ADCn inter-observer** |  |  |
| **Individual measures** | 0.784(0.628-0.880) | 0.787(0.632-0.881) |
| **Average measures** | 0.879(0.771-0.936) | 0.887(0.775-0.937) |
| **rADC inter-observer** |  |  |
| **Individual measures** | 0.802(0.656-0.890) | 0.781(0.603-0.882) |
| **Average measures** | 0.890(0.792-0.942) | 0.877(0.752-0.937) |

**Supplementary Table 6.** The performance of each machine learning models in the study set.

| **Model** | **AUC** | **Se** | **Sp** | **Accuracy** | **F1 score** |
| --- | --- | --- | --- | --- | --- |
| **Logistic Regression** | 0.897 | 0.805 | 0.888 | 0.824 | 0.824 |
| **SVM** | 0.876 | 0.816 | 0.865 | 0.824 | 0.824 |
| **NB** | 0.863 | 0.839 | 0.764 | 0.790 | 0.790 |
| **Ensamble** | 0.892 | 0.816 | 0.854 | 0.807 | 0.807 |

Abbreviations: SVM = support vector machine, NB = Naive Bayes, AUC = The area under the curve, Se = sensitivity, Sp = specificity
